# Editorial: Love Island: Gender, Sexuality and Intimate Relationships

**DOI:** 10.3389/fsoc.2021.781970

**Published:** 2022-01-17

**Authors:** Sophie Woodward, Laura Fenton

**Affiliations:** Sociology, The University of Manchester, Manchester, United Kingdom

**Keywords:** reality television, gender, media, authencity, culture

Love Island has become a shorthand for the vacuous ills of celebrity, fakeness, disposable fashion and narcissism; these characterisations overlook the internal conflicts, tensions and complexities of the programme. A reality television programme where participants live together and form romantic heterosexual couplings to compete for the possibility of lucrative sponsorships contracts, media work and a cash prize, Love Island has an arresting appeal: viewing figures for the final episode of 2021 were 2.8 million (Variety, 2021). We argue that Love Island is sociologically fascinating for several reasons, not least that viewers become invested in the contestants while simultaneously feeling unease, disgust, and voicing criticisms of the programme. Viewers observe and get involved in the relationships portrayed on the programme and in the process reflect upon their own relationships and the ideal relationship more broadly. They often become active and vocal observers of relationships, with concepts such as ‘‘gaslighting’, ‘toxic masculinity’, and racist double standards featuring heavily in how the programme is discussed on the social media platform Twitter. In short, Love Island turns the ‘stuff’ of private troubles into public problems.

In the process of making intimate issues topics for collective discussion, the programmer’s producers use tactics such as editing and deception. Despite upgraded ‘duty of care’ protocols, the programme repeatedly challenges couples, and has been accused of emotionally manipulating them. As Helen Wood (2021) has commented, ‘duty of care’ procedures and a focus on online trolling risk individualising and de-politicising the harms endured by contestants by shifting attention away from the ethics of production. As a ‘post-broadcast regulator’, Ofcom is not permitted to “interfere in programme-making beyond its remit to protect the public from ‘unfair treatment’”. For Wood, the ethics of working practices in reality television and care for participants require greater scrutiny (Wood, 2021). In relation to working conditions, the show is arguably at its worst when emotional displays—particularly tears and expressions of jealously or hurt—are used as the ‘money shot’ (Allen, 2018). For example, across several series misleading footage and photographs taken during the infamous ‘Casa Amor’ episodes (where male contestants move into a new Villa and more contestants are brought in to challenge existing relationships) have been shown to contestants, causing considerable distress.

As viewers we are always aware these are not ‘real’ relationships as the heavy editing of the programme is apparent, with staged ‘chats’ about feelings or ‘where your head is at’. Vocabulary that appeared in previous series, appears to be fed back to new contestants by producers. The question ‘what’s your type?’ - repeated dozens of times over Love Island’s eight series—became arguably too central to conversations in the current series. The question ‘shall we have a chat?’ also emerged several times an episode making manifest the staging of the programme. When conversations appear overly staged and devoid of spontaneous content, the show’s status as ‘advertainment’ becomes all the more obvious and irksome. The relations between fakeness and reality are fascinating, as contestants have to juggle their performances so as to not to seem too earnest or contrived, striving for authenticity. Viewers question whether some participants have gone on the show for a fashion career (a clothing range in Pretty Little Thing for example) when they are unconvinced of the authenticity of the displays of feelings towards others.

Consistent with the recent critiques discussed above, this special Issue engages sociologically with Love Island. L’Hoiry explores how the programme’s embracing of social media appears to have had unintended consequences. While the strategy of encouraging multi-platform consumption has enabled the programme to capitalise on opportunities for monetization, the show’s strategic engagement with platforms like Twitter has also led to an intense sense of investment on the part of audiences. This investment can breed a sense of ownership, with fans adopting a shared responsibility for holding producers to account. L’Hoiry explores examples of accusations of manipulation circulating on Twitter during the 2018 series. Though accusations of pre-determined outcomes, fakery and stage-management may eventually undermine the show’s appeal, for now “rather than switching off altogether, Love Island fans instead seek to consume the show on their own terms, binding together to re-craft narratives when the content presented to them by producers does not meet with the audience’s approval”.

A core issue of the other three articles is gender and an exploration of masculinity in particular. Nichols’ article explores the possibilities and limitations of theories of hegemonic masculinity in relation to two seasons of Love island (2017 and 2018) to explore both the ways dominant forms of masculinity are reinforced, but also how diverse masculinities are performed. Denby’s article develops the critique of masculinity in relation to the show’s brand of heterosexuality by centring the impact of the heteronormative focus of the programme has on women and the depiction of female contestants. Denby takes case studies form Love Island (series 2016–2020) to examine gender roles in contemporary intimate relationships arguing that Love Island perpetuates outmoded heteronormative ideals. Drawing on their experience of running relationship education programs and their research with young people on their perceptions of Love Island, Porter and Standing examine the janus-faced nature of show: on the one hand it can be seen to reinforce normative heterosexuality and to even normalize emotional abuse, while on the other hand it acts as a catalyst for discussion and creates space for challenging dominant constructions of relationships. In the art-based workshops and focus group discussions conducted by the authors, young people questioned the ‘ordinariness’ and authenticity of contestants and their relationships. However, many appreciated the opportunities that it opened for talking about healthy and unhealthy relationships. Given the inadequacy of current relationships education in the UK context, the authors explore the potential for the programme to improve this area of the curriculum.

